# Supplementary material for: Structural Preferences Shape the Entropic Force of Disordered Protein Ensembles
Source: J Phys Chem B. 2023 May 8;127(19):4235–44. doi: 10.1021/acs.jpcb.3c00698 (PMC10201532; doi:10.1021/acs.jpcb.3c00698)
Supplement: Supplementary file 1 — jp3c00698_si_001.pdf [file jp3c00698_si_001.pdf]

Supporting Information for Publication:

Structural Preferences Shape the Entropic Force of Disordered Protein Ensembles

Feng Yu[1] and Shahar Sukenik[1,2,*]

1. Quantitative Systems Biology Program, University of California, Merced, California, United States, 95343
2. Department of Chemistry and Biochemistry, University of California, Merced, California, United States, 95343

* correspondence to: ssukenik@ucmerced.edu

Figure S1

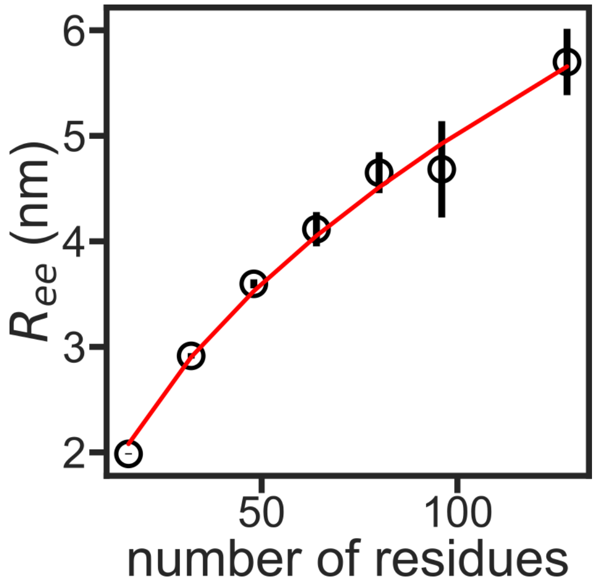

Figure S1. GS repeats match homopolymer scaling law under buffer conditions. The average end-to-end distance from five repeats vs the total number of residues for a series of Gly-Ser repeats, The error bars are the standard deviation of the five repeats. The red curve is the result of fitting to $R_{ee} = R_0 N^{\nu}$, with $R_0 = 0.55 \pm 0.06$ nm and $\nu = 0.48 \pm 0.03$. Errors are obtained from the fit.

Figure S2

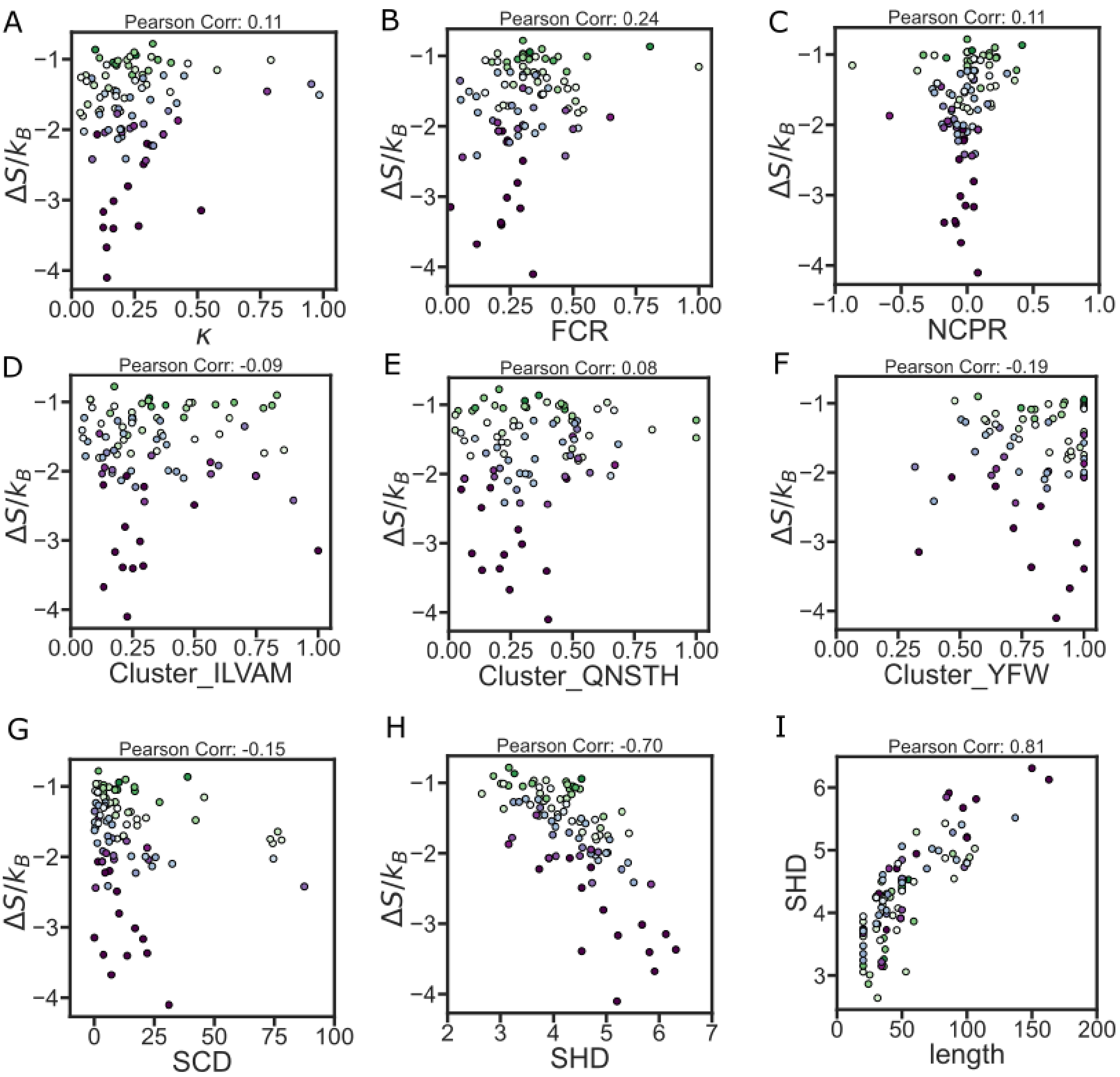

Figure S2. Sequence features correlated with the entropic force strength. The entropic force strength is plotted vs several sequence features. Unless stated otherwise, all sequence features are calculated using the localCIDER python package. (**A**) κ: a metric for mixing of charged amino acids[18] (**B**) FCR: fraction of charged residues, (**C**) NCPR: net charge per residue, (**D**) Cluster_ILVAM: hydrophobic amino acid mixing calculated using the same algorithm as κ, (**E**) Cluster_QNSTH: polar amino acid mixing calculated using the same algorithm as κ, (**F**) Cluster_YFW, aromatic amino acid mixing calculated using the same algorithm as κ. (**G**) SCD: sequence charge decoration[19] (**H**) SHD: sequence hydropathy decoration[21] (**I**) SHD vs sequence length shows a strong correlation with the sequence length. This may explain at least some of the correlation with entropic force, which is also shown to correlate with sequence length (**Fig. 5A**).

Figure S3

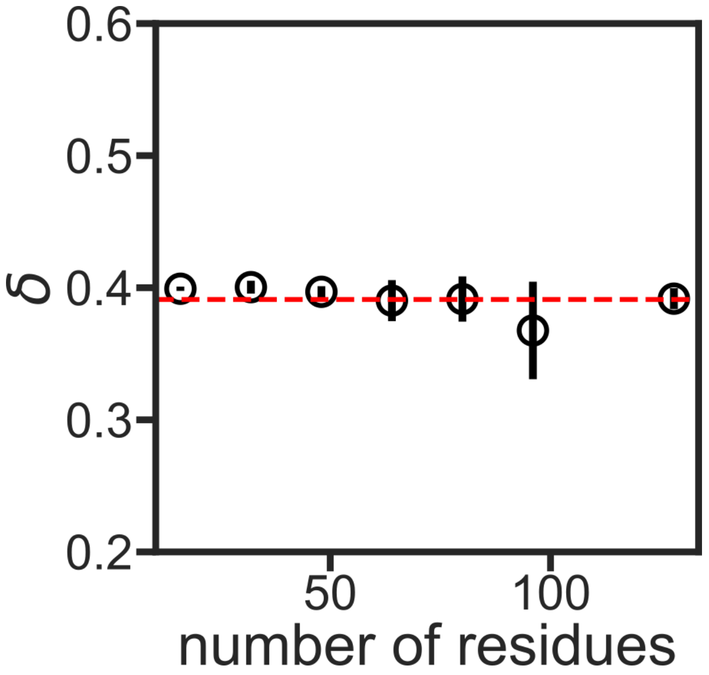

Figure S3. GS repeat asphericity is independent of length. The average asphericity of GS repeats vs the number of residues in the sequence. The mean of all seven data points is shown by the red line, with $\delta = 0.39 \pm 0.01$.

Figure S4

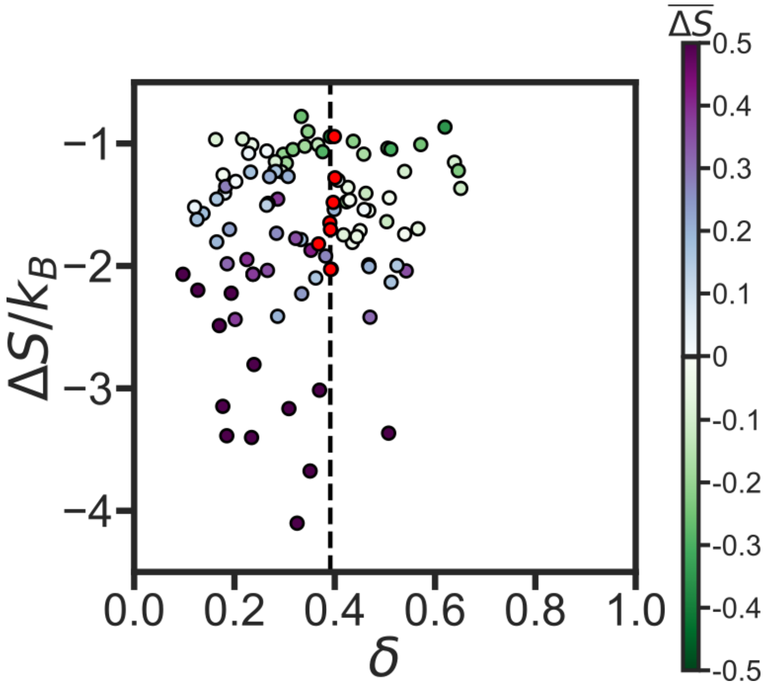

Figure S4. Entropic force as a function of average asphericity. The black line represents the length-independent asphericity of GS repeats shown in **Fig. S3**. Each marker represents a single IDR, color-coded as in **Fig. 5A** , with stronger purple (green) markers showing a stronger (weaker) entropic force compared to the GS repeat of the same size.
